# Supplementary figures and images for: Efficacy evaluation of the S-adenosylhomocysteine hydrolase inhibitor MSD-914 in rhesus macaques (Macaca Mulatta) challenged with Ebola virus by the intramuscular route
Source: PLoS One. 2026 Feb 6;21(2):e0340118. doi: 10.1371/journal.pone.0340118 (PMC12880677; doi:10.1371/journal.pone.0340118)

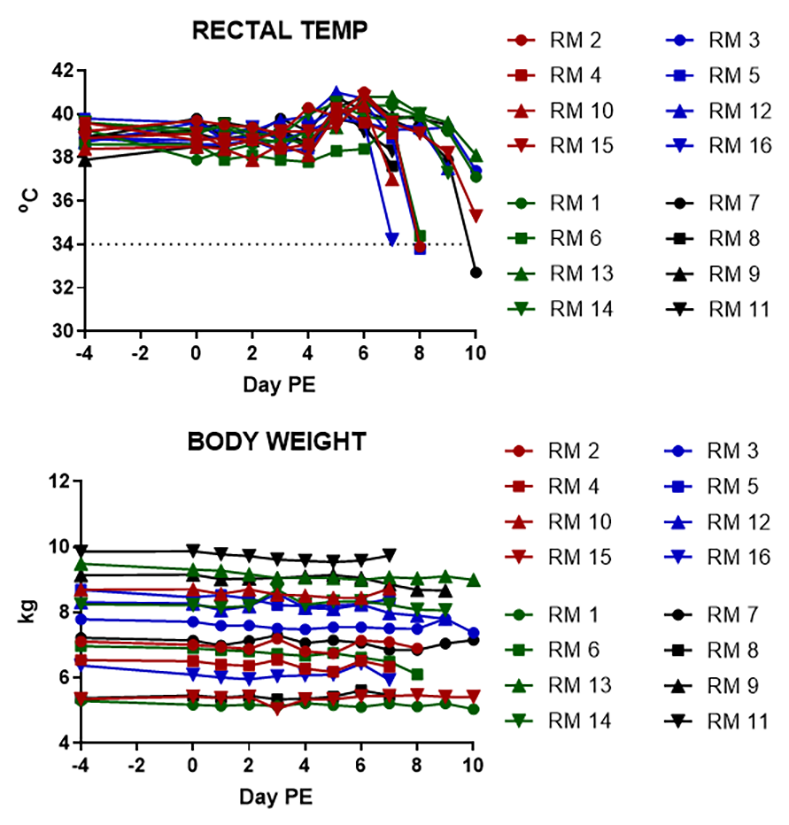

Supplement: S1 Fig — Red lines and symbols represent Group 1 (0.8 mg/kg), green lines and symbols represent Group 2 (0.27 mg/kg), blue lines and symbols represent Group 3 (0.09 mg/kg), and black lines and symbols represent Group 4 (controls). RM = rhesus macaques, PE = postexposure. (TIF) [file pone.0340118.s001.tif]

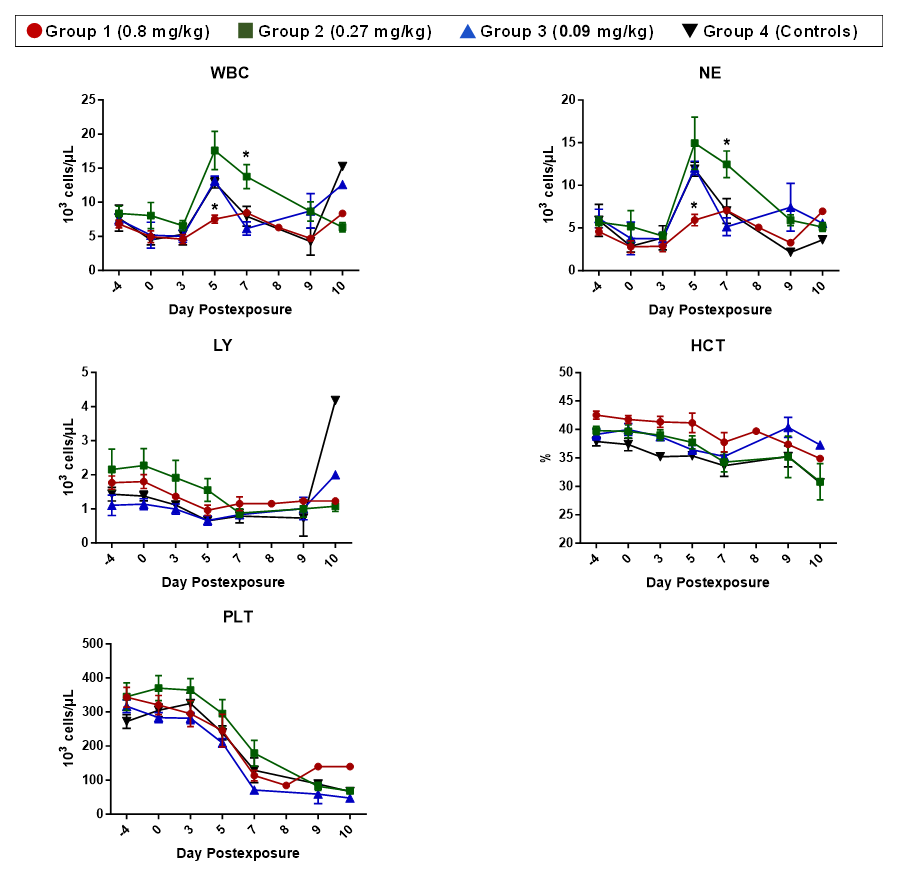

Supplement: S2 Fig — Hematology was performed on a Beckman Coulter DxH520 hematology analyzer. Grouped data are shown, with error bars depicting the SEM. Red lines and symbols represent Group 1 (0.8 mg/kg), green lines and symbols represent Group 2 (0.27 mg/kg), blue lines and symbols represent Group 3 (0.09 mg/kg), and black lines and symbols represent Group 4 (Controls). Statistical significance (p < 0.05) is depicted by the *. (TIF) [file pone.0340118.s002.tif]

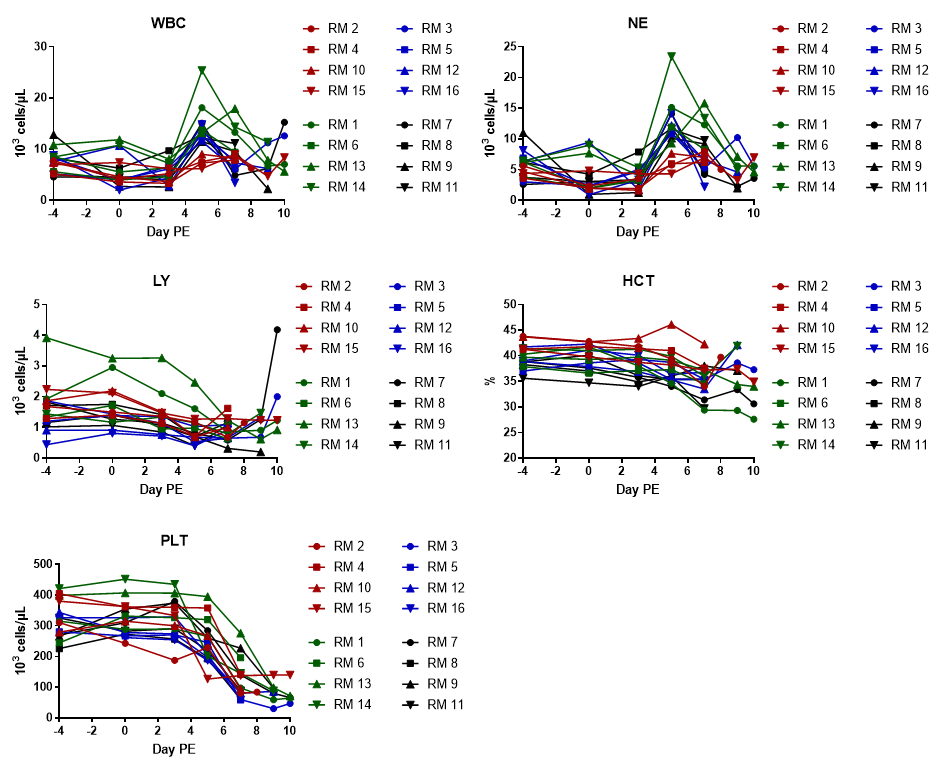

Supplement: S3 Fig — Red lines and symbols represent Group 1 (0.8 mg/kg), green lines and symbols represent Group 2 (0.27 mg/kg), blue lines and symbols represent Group 3 (0.09 mg/kg), and black lines and symbols represent Group 4 (controls). RM = rhesus macaques, PE = postexposure. (TIF) [file pone.0340118.s003.tif]

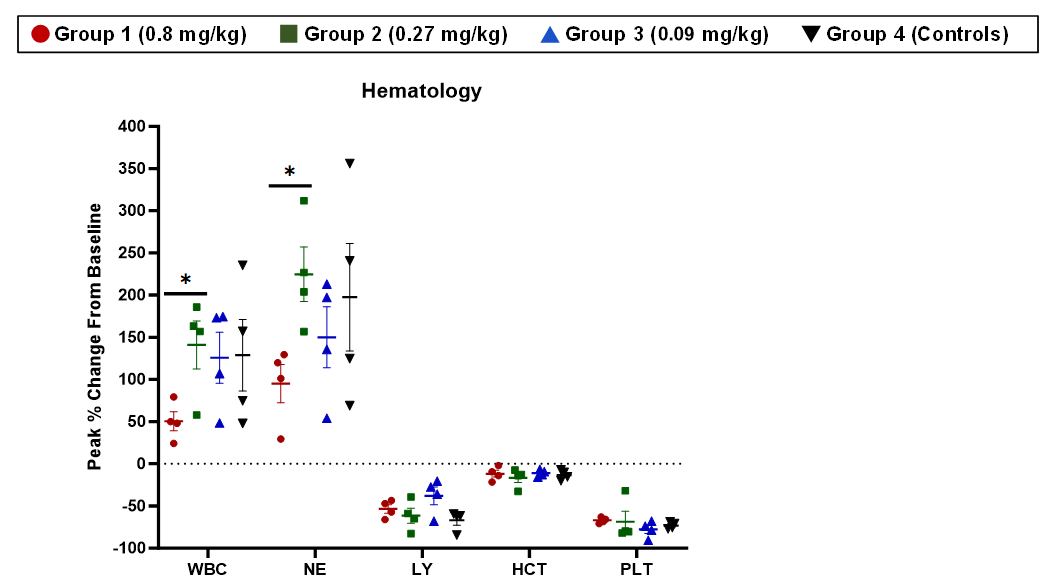

Supplement: S4 Fig — The % change from baseline for peak values is shown. For WBC, NE, and all clinical chemistry parameters, the peak is the highest value obtained. For LY, HCT, and PLT, the peak is the lowest value obtained. Symbols represent individual animals, and the horizontal line is the group mean. Error bars represent the SEM. Statistical significance (p < 0.05) is depicted by the *. Red lines and symbols represent Group 1 (0.8 mg/kg), green lines and symbols represent Group 2 (0.27 mg/kg), blue lines and symbols represent Group 3 (0.09 mg/kg), and black lines and symbols represent Group 4 (Controls). (TIF) [file pone.0340118.s004.tif]

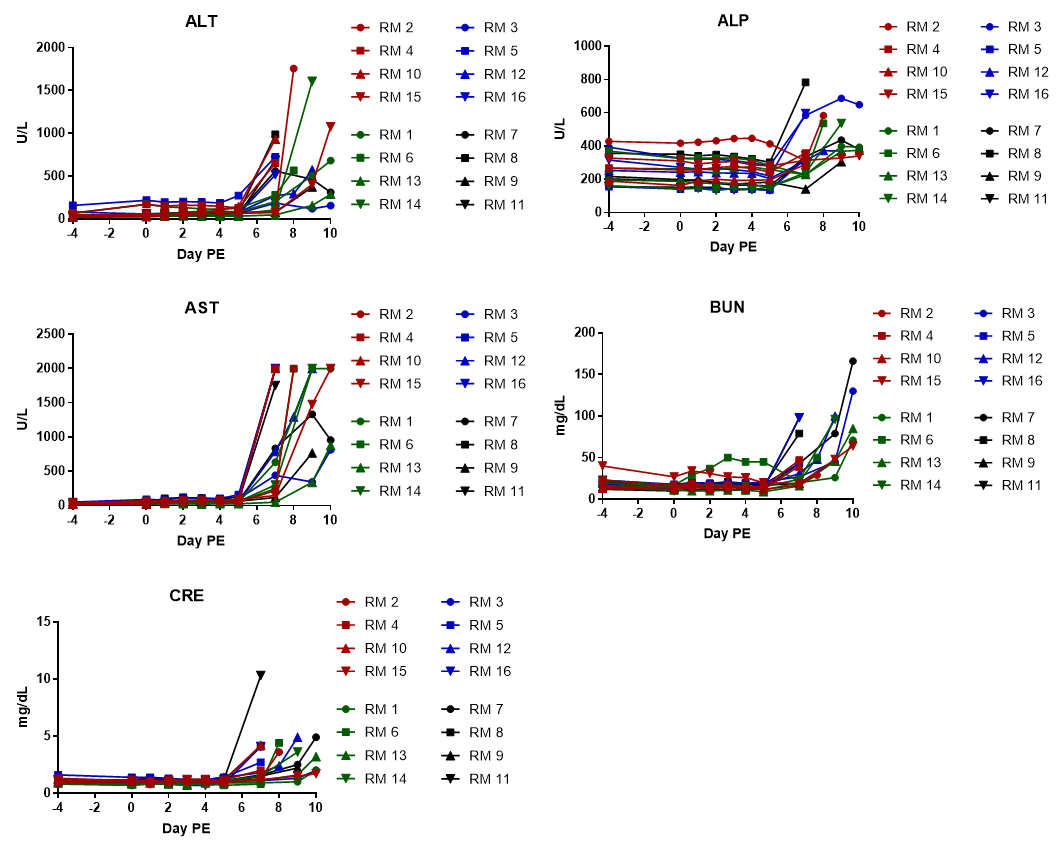

Supplement: S5 Fig — Red lines and symbols represent Group 1 (0.8 mg/kg), green lines and symbols represent Group 2 (0.27 mg/kg), blue lines and symbols represent Group 3 (0.09 mg/kg), and black lines and symbols represent Group 4 (controls). RM = rhesus macaques, PE = postexposure. (TIF) [file pone.0340118.s005.tif]
