# Supplementary material for: Efficacy evaluation of the S-adenosylhomocysteine hydrolase inhibitor MSD-914 in rhesus macaques (Macaca Mulatta) challenged with Ebola virus by the intramuscular route
Source: PLoS One. 2026 Feb 6;21(2):e0340118. doi: 10.1371/journal.pone.0340118 (PMC12880677; doi:10.1371/journal.pone.0340118)
Supplement: Attachment — Submitted filename: Response to Reviewers.docx [file pone.0340118.s009.docx]

Dear Editor,

The authors would like to Thank the Editors and the Reviewers for their comments and useful suggestions. Below are our revisions and edits to both the Editors and Reviewers comments

**Responses to Editors Comments**

1. *Please ensure that your manuscript meets PLOS ONE's style requirements, including those for file naming.* <https://journals.plos.org/plosone/s/file?id=wjVg/PLOSOne_formatting_sample_main_body.pdf> and

<https://journals.plos.org/plosone/s/file?id=ba62/PLOSOne_formatting_sample_title_authors_affiliations.pdf>

Response: The authors have revised and made editorial changes to meet the manuscript requirements including style requirement and file naming.

2. *We note that your study design may include death of a regulated animal as a likely outcome or planned experimental endpoint. At this time, we request that you please report additional details in your Methods section regarding animal care and use for the survival study, as per our editorial guidelines* (<http://journals.plos.org/plosone/s/submission-guidelines#loc-humane-endpoints>). For easy reference, we have attached a checklist that may be relevant for your submission. Please complete all items on the checklist at the following link: <http://journals.plos.org/plosone/s/file?id=bb1d/plos-one-humane-endpoints-checklist.docx> Please upload the completed checklist as file type “Other” when resubmitting your manuscript. This document is for internal journal use only and will not be published if your article is accepted. We very much appreciate your attention to these requests and support of improved reporting standards in PLOS ONE submissions

Response: Requirements for humane endpoints from the link above are listed below, with their locations in the manuscript:

1. The specific criteria (i.e. humane endpoints) used to determine when animals should be euthanized:

Response: See pages 7-9 for information related to criteria for euthanasia for NHPs, and page 9 for information related to criteria for euthanasia for mice.

2. The duration of the experiment:

Response: This information can be found in Table 3 for the mice. For clarity, we have also added the end-of-study day (Day 21) to the methods section on page 9. For NHPs, this information can be found in page 26. Due to a lack of efficacy and for humane reasons for remaining animals, the study was terminated on Day 10. For clarity, we have added the end-of-study day to the methods section on page 9.

3. The numbers of animals used, euthanized, and found dead (if any); the cause of death for all animals:

Response: The number of animals on study can be found on page 6 in the methods section, and in tables 3 and 6. All animals that met criteria for euthanasia died from advanced EBOV disease, as is described in the results section. Information related to numbers of animals found dead vs euthanized cannot be presented as this is sensitive information that the IACUC will not allow to be disclosed in open print.

4. How frequently animal health and behavior were monitored:

Response: This information is provided on page 9 for mice, and pages 7-8 for NHPs.

5. All animal welfare considerations taken, including efforts to minimize suffering and distress, use of analgesics or anaesthetics, or special housing conditions: Response: Information related to husbandry, animals on study, humane endpoints, the use of analgesics, and euthanasia are discussed on pages 6-9.
3. *Thank you for stating the following financial disclosure: “This project was supported by Defense Threat Reduction Agency (DTRA) under project Number CB11160.” Please state what role the funders took in the study.  If the funders had no role, please state: "The funders had no role in study design, data collection and analysis, decision to publish, or preparation of the manuscript." If this statement is not correct you must amend it as needed. Please include this amended Role of Funder statement in your cover letter; we will change the online submission form on your behalf*.

Response: The funders had no role in study design, data collection and analysis, decision to publish, or preparation of the manuscript.

4. We note that your Data Availability Statement is currently as follows: “All relevant data are within the manuscript and in Supporting Information files.”

Please confirm at this time whether or not your submission contains all raw data required to replicate the results of your study. Authors must share the “minimal data set” for their submission. PLOS defines the minimal data set to consist of the data required to replicate all study findings reported in the article, as well as related metadata and methods (<https://journals.plos.org/plosone/s/data-availability#loc-minimal-data-set-definition>).

For example, authors should submit the following data:

- The values behind the means, standard deviations and other measures reported;

- The values used to build graphs;

- The points extracted from images for analysis.

Authors do not need to submit their entire data set if only a portion of the data was used in the reported study.

If your submission does not contain these data, please either upload them as Supporting Information files or deposit them to a stable, public repository and provide us with the relevant URLs, DOIs, or accession numbers. For a list of recommended repositories, please see <https://journals.plos.org/plosone/s/recommended-repositories>.

If there are ethical or legal restrictions on sharing a de-identified data set, please explain them in detail (e.g., data contain potentially sensitive information, data are owned by a third-party organization, etc.) and who has imposed them (e.g., an ethics committee). Please also provide contact information for a data access committee, ethics committee, or other institutional body to which data requests may be sent. If data are owned by a third party, please indicate how others may request data access.

Response: All RAW data associated with the Figures and Table 2 have been provided as supporting information.

**Response to Reviewer’s Comments**

Reviewer #1: The study by Johnston et al. evaluated the efficacy of the S-adenosylhomocysteine hydrolase inhibitor MSD-914 in vitro against Ebola, Sudan and Marburg virus and against MA-EBOV in mice and Ebola in rhesus macaques. Two-digit nM EC_50_ were achieved in various cells lines and 100% protection was observed in a lethal mouse model above a certain dose threshold. This was used to justify an escalating dose efficacy study in rhesus macaques, where near the MTD, efficacy was not observed. It would also be useful to indicate the in vitro EC90 or 99 values if these are available, as for NHPs in filovirus challenges it is unlikely that achieving the EC50 will be effective as a therapeutic.

Response: Below is the Table with details about the EC_50_, EC_90_ and SI values for different filoviruses and cell types. The Table is also included in the Results section of the Manuscript as revised Table 2

| **Cell Type** | **Pathogen** | **EC50 [µM]** | **EC90 abs [µM]** | **CC50 [µM]** | **SI** |
| --- | --- | --- | --- | --- | --- |
| MRC-5 | EBOV | 0.027 | 0.35 | >30 | >1111 |
| HeLa | EBOV | 0.064 | 0.43 | >25 | >390.6 |
| HFF | EBOV | 0.065 | 0.50 | >25 | >384.6 |
| HeLa | MARV | 0.071 | 0.58 | >25 | >352.1 |
| HeLa | SUDV | 0.055 | 1.11 | >25 | >454.5 |

Perhaps discussion on altered dosing (more than once per day, and whether that would be tolerated), given the fast clearance – while acknowledging the difficulty of performing such studies in NHPs in CL4 would be worthwhile. Also, the daily dosing of ketamine in NHPs to administer oral drugs is likely problematic to overall animal health and potential confounding factor.

Response: The following sentences have been added in the discussion section. “Current data indicate that even with a twice-daily (BID) dosing regimen at 0.8 mg/kg, plasma concentrations cannot be maintained above the EC50. To achieve sustained plasma levels above the EC50, a three-times-daily (TID) dosing regimen may be necessary, but this presents significant logistical challenges in a BSL-4 laboratory setting, particularly concerning animal welfare and the feasibility of administering multiple doses daily. While a single daily (SID) or BID dosing regimen at 1.6 mg/kg may offer a better chance of maintaining plasma concentrations above the EC50, tolerability and PK of this higher dose in non-human primates (NHPs) must first be confirmed”.

Minor comments.
Administration to RM of treatment was daily oral. This suggests animals underwent anesthesia daily? Was this delivered by gavage/gastric tube? If no, how was appropriate delivery ensured. How was the performed in mice? By oral gavage? Or some other route. Please add more details. Oral gavage is mentioned later in the results, but this should also be specified in the M&M.

Response: The drug was administered via oral gavage in mice and NHPs. This has now been clearly stated in the Materials and Methods sections

Figure 1b. Percent sick animals is an unusual metric. Perhaps showing clinical score or considering an alternative Y axis title. Percent animals with clinical score. Would seem more appropriate. The descriptions in the text should also be modified accordingly.

Response: The Y axis is appropriate as stated. For mice, we assess animals in a group and list them as either healthy, sick, moribund, or deceased. So % sick animals is the % of the animals in a group that were sick at the time of an observation. Therefore, no update is required.

The lack of difference between groups in the mouse study in terms of viral RNA is surprising. Was infectious virus also evaluated? Were any tissues analyzed in animals that were sampled on day 6?

Response: Plaque assays to assess infectious virus in blood and tissues were not performed on this study.

If EC50 values are not reached or maintain regardless of dosing in mice, but the compound is effective, is a non-direct antiviral effect suspected? This could be discussed.

Response: The following sentences have been added in the discussion

The effectiveness observed, despite EC50 values not being reached or maintained, suggests the potential for a non-direct antiviral effect. The compound has reported activity against at least one non-direct antiviral target, AHCY. Additionally, while we did not explore the metabolites generated in mice, we cannot exclude the possibility that a metabolite derived from the parent compound could exhibit more potent inhibition of Ebola, thereby contributing to the overall efficacy.

Perhaps a line indicating the EC50 value could be included in the PK graphs?

Response: The average EC50 values of 71 nM and 55 nM have been converted to ng/mL using a molecular weight of 252, and these values have been incorporated into the PK graphs (**Figure 4A and 4B**).

Given tolerability issues at 3mg/kg in RM and acceptable profile at 0.8mg/kg, there is not much room increase the dose to see if a higher dose would be effective, although it is possible that a 1.6mgkg dose would be tolerated.

Response: We agree with the reviewer that we cannot test a higher dose to determine if it would be effective.

Figure 6 and 7, 9 could be moved to supplemental. No critical data is included in these figures.

Response: The authors would like to Keep Figure 9 and move Figures 6 and 7 to supplementary. Hence figure numbers have now been appropriately revised

There appears to be inconsistent error bars in figure 8. Some points or groups do not seem to have error bars, which seems unlikely for clinical chemistry data. Especially as group 1 ALT data seems erratic (early spike in ALT, possibility due to drug toxicity concomitant with EBOV infection?), but no error bar in the samples?

Response: Some of the datapoints in Figure 8 do not have any error bars as there is only one animal that was alive at the timepoints when the samples were collected. Hence no error bars could be generated.

The lack of infectious virus titers in the RM is unfortunate. The explanation given is also not typical of that observed by other. High viral titers in the blood can usually be observed. Possibly day 5 was too early? Were viral titers in a key organs, such as the liver assessed at necropsy. While likely equivalent due to the outcome, was this investigated?

Response: Issues relating to fibrin clots in the serum are discussed on page 35. We have added a statement to this section to clarify that fibrin clots were present in the serum samples processed for plaque assay, and this directly affected the performance of the plaque assay. In regards to tissues, histology and IHC were performed to demonstrate pathologic changes and the presence of viral antigen (see Tables 8-9, and Figures 9-10). PCR and plaque assay were not performed on tissue sections collected on this study.

**Reviewer #2:** The authors provide a detailed description of the therapeutic administration of MSD-914 in Ebola virus challenge models using both balb/C mice and Rhesus macaques. Interestingly, while the compound provided strong protection against lethal disease in mice at multiple concentrations, no efficacy was seen in the nonhuman primate model of infection.
The study is interesting and highlights an important consideration for animal models of Ebola virus disease where small or large animals can demonstrate divergent responses to the same treatment across multiple animal models. Here, the authors provide an open description of these divergent results. While this study is interesting, there were several considerations identified for the authors to expand on the in the Results and Discussion sections of the manuscript. In particular, greater discussion on the clear efficacy differences between the two models should be expanded on. These will be discussed below:

Introduction
1. Regarding the statement on the CFR being up to 90% for EVD, technically yes, but those outbreaks were among the earliest recorded. It would be recommended to use the more recent 40-60% CFRs presented in reviews or other reports that are based on overall cases across all outbreaks.

Response: Appropriate edits have been made in the Introduction

2. When describing the countries impacted by EVD during the 2013-2016 epidemic, I would actually recommend Nigeria and Mali be included here as well given that both countries had higher case numbers as compared to non-African countries. It's more to ensure that the impact on these countries is not forgotten in the literature

Response: Appropriate edits have been made in the introduction

3. While the first part of the introduction focuses heavily on the 2013-2016 EVD epidemic, I would recommend highlighting the recurrent outbreaks in DRC during the last decade as well as the two outbreaks associated with SUDV in Uganda. The increasing frequency of these outbreaks as well as the relation to both zoonotic spillovers as well as new chains of transmission due to persistent infection highlight the need for MCMs, etc.

Response: Additional information added to the introduction related to recent outbreaks.

4. The paragraph on therapeutic studies and animal models should be heavily referenced as there's a lot of material. It would also seem prudent to perhaps describe the models - do mice recapitulate human disease, what is typically seen, etc. Same as the prior comment for NHPs - references and a bit of description on natural history studies in these animals. In the last sentence, the type of mice and species of NHPs used in the investigation should be introduced here.

Response: Additional information added.

5. There's been a recent taxonomy update for filoviruses that CDC is now using on their webpages so should likely ensure that the manuscript adopts a similar naming strategy: <https://ictv.global/report/chapter/filoviridae/filoviridae/orthoebolavirus>

Response: Relevant edits have been made.

Mats/Methods
1. How was this MOI optimized (mentioned in Mats/Methods) and this should be described in the results.

Response: The multiplicity of infection (MOI) for each virus stock is optimized by first performing a rainbow infection wherein cells are infected with varying MOI of the virus and plates fixed in 10% formalin at time points of 48 or 72 hours. Infection rate is then quantitated using image-based immunofluorescence assay using viral specific antibodies. The MOI and time that gives an infection rate of 50-75% are selected and further confirmed by a second round of infection studies. These optimal conditions are used for therapeutic screening.

In the material and methods selection, we have added the MOI that were used for each virus/cell type combination.

2. Can the authors provide any additional comment(s) on the difference in controls between the different species? Was there a reason for not using 0.5% methylcell as the treatment control in mice given that the treated animals would have received this carrier throughout dosing

Response: For the vehicle control group we used 0.5% methycellulose diluted in PBS. This has been corrected in the revised version of the manuscript.

**Results**
1. For the in vitro studies described in the first Results section, this data should be presented as a table or figure in the text

Response: Data is presented in a Table format in the results section (Revised Table 2)

| **Cell Type** | **Pathogen** | **EC50 [µM]** | **EC90 abs [µM]** | **CC50 [µM]** | **SI** |
| --- | --- | --- | --- | --- | --- |
| MRC-5 | EBOV | 0.027 | 0.35 | >30 | >1111 |
| HeLa | EBOV | 0.064 | 0.43 | >25 | >390.6 |
| HFF | EBOV | 0.065 | 0.50 | >25 | >384.6 |
| HeLa | MARV | 0.071 | 0.58 | >25 | >352.1 |
| HeLa | SUDV | 0.055 | 1.11 | >25 | >456.5 |

2. Regarding the 30% of the animals in Group 2 that became sick while none in Groups 3 & 4 showed the same pattern, can the authors provide some context or insights on why this may have occurred?

Response: It is possible that higher dose of the drug (Group 2 mice) in combination with infection may have contributed to the animal becoming moribund or sick.

3. In Figure 3 (and corresponding Results description), viral genomes were identified across all groups at Day 6 PE. There are a couple of considerations here. One, did the authors look at viral loads across different major organs to better understand if virus dissemination was altered between the treatment and control groups. Second, it would be worthwhile to assess whether infectious virus was present or whether whole genomes were present across the groups.

Response: Plaque assays to evaluate infectious virus in tissues were not performed, as qPCR data from the plasma samples of mice infected with maEBOV did not show any differences in viral RNA.

4. Are there any inferences from the authors on why the Group 1 and Control Group animals had the highest severity score in the histology data? Was there any indication that this might have been due to some synergistic or additive effect of the treatment and infection? It looks as if Group 1 was higher across both the liver and inguinal lymph node columns. This also seemed to correlate with the immunohistochemistry scoring in Table 8. Can the authors provide some additional comments on this?

Response: Although it is possible that there was an additive effect of treatment in the presence of infection, it needs to be understood that scoring of tissues is determined by the perceived percentage of the tissue section affected. This means that there is a bit of subjectivity wherein the difference between a tissue being called a 3 (25-50% affected) and a 4 (50-75% affected), for example, could be negligible (in other words, a determination of 52% of a tissue affected would be a 4, but a determination of 48% of a tissue affected would be a 3, and 50% of the tissue affected could be graded a 3 or a 4). In addition, the section of tissue being evaluated could affect overall scoring. So typically we would look for much larger differences per tissue than what we are seeing in this study, not necessarily the overall score as this is a reflection of the additive differences which could be more greatly affected by subjectivity as described above.

Discussion
1. I would suggest that calling these "human" EBOV is a bit of a misnomer as compared to wild-type as there's no data to suggest an adaptation towards a humanized strain or phenotype

Response: The word “human’ has been deleted and replaced with “wild type” Ebola virus

2. The third paragraph of the Discussion should be expanded as well as referenced. For example, are there differences in virus distribution, symptomology, etc? Would recommend further expansion on the similarities between Group 1 and the control group (Group 4) in regard to severity scores - why might these have scored similarly as compared to Groups 2 and 3? Also, it would be recommended to use consistent naming for Group 4 throughout all tables and figures for the NHP experiments as the labeling swaps between Controls and Group 4

Response: Although it is possible that there was an additive effect of treatment in the presence of infection, it needs to be understood that scoring of tissues is determined by the perceived percentage of the tissue section affected. This means that there is a bit of subjectivity wherein the difference between a tissue being called a 3 (25-50% affected) and a 4 (50-75% affected), for example, could be negligible (in other words, a determination of 52% of a tissue affected would be a 4, but a determination of 48% of a tissue affected would be a 3, and 50% of the tissue affected could be graded a 3 or a 4). In addition, the section of tissue being evaluated could affect overall scoring. So typically we would look for much larger differences per tissue than what we are seeing in this study, not necessarily the overall score as this is a reflection of the additive differences which could be more greatly affected by subjectivity as described above. As far as labeling swaps, we have updated any figures that only identified the group as controls to add the appropriate “Group 4” label for clarity.
