# Supplementary material for: Efficacy evaluation of the S-adenosylhomocysteine hydrolase inhibitor MSD-914 in rhesus macaques (Macaca Mulatta) challenged with Ebola virus by the intramuscular route
Source: PLoS One. 2026 Feb 6;21(2):e0340118. doi: 10.1371/journal.pone.0340118 (PMC12880677; doi:10.1371/journal.pone.0340118)
Supplement: Attachment — Submitted filename: Author Response 01Dec2025_.docx [file pone.0340118.s010.docx]

17 November 2025

Dear Editor,

The authors would like to Thank the Editors and the Reviewers for their comments and useful suggestions. Below are our revisions and edits to both the Editors and Reviewers comments

Reviewer #3: In this manuscript “Efficacy evaluation of the S-adenosylhomocysteine hydrolase inhibitor MSD-914 in rhesus macaques (Macaca Mulatta) challenged with Ebola virus by the intramuscular route” by Johnston et al the authors present an evaluation of the small molecule therapeutic MSD-914 for the treatment of filovirus infections, including in vitro evaluation against Ebola, Marburg and Sudan viruses, in depth in vivo pharmacokinetics, successful treatment of mice infected with MA-EBOV above a threshold dose, and documentation of failure of efficacy in when treating NHPs with Ebola virus. The experiments reported are well designed and presented, and provide convincing evidence for the authors conclusions. The differences between the in vitro, murine and NHP data are remarkable. This paper serves as an excellent example of the importance of high-fidelity animal models particularly for viral hemorrhagic fevers. Minor changes to the manuscript may strengthen particular points

1. **Reviewer’s Comment:**

Minor comments:
Line 16: The current outbreak (as of Oct 2025) in the DRC of EVD is ongoing since the original submission of this article and mortality figures can be updated to 64 confirmed or probable cases and 43 deaths.

Author Response: Authors have updated this section to include the revised numbers

2. **Reviewer’s Comment**:

Line 44: This section of the introduction contains results.

Author Response:	The results portion has been removed and the paragraph revised.

3. **Reviewer’s Comment**:

Line 312: A plaque assay or similar (e.g. TCID50) would have provided additional insight into the mechanism for survival despite equivalent viremia. As the authors note this was not performed, and a discussion on whether this could account for survival despite equivalent viremia (and the inability to perform plaque assays on the NHP experiments for additional insight) addressed in the discussion section as a limitation.

Author Response: We agree with the reviewer that a plaque assay would have provided additional insight into the mechanism of action. However, due to lack of sufficient sample to perform these studies was a limitation.

4. **Reviewer’s Comment**:

Line 550, Line 573: Statistical test for differences in histology on liver findings given overall milder presentation of disease by multiple measures should be performed, as the authors address numerical differences in one group. Although samples sizes may be too small to find significance (e.g. a Kruskal-Wallis test on the summed scores provided does not show significance) this should be done for completeness.

Author Response: The reviewer’s comment is appreciated. However, due to the sample size and the subjectivity of histologic scoring which is meant more for observational relevance rather than statistical significance, performing statistical analyses on these types of measures would not likely yield sufficient information to support any additional meaningful conclusions related to group differences. Therefore, these analyses will not be performed for this study.

5. **Reviewer’s Comment**:

Line 585: If the compound has activity against host targets this should be cited or documented experimentally.

Author Response: The AHCY inhibitory activity of MSD-914 is mentioned earlier in the manuscript, on Line 36, and is cited there in the text (Reference 15). Appropriate edits have been made in this section and references cited.

6. **Reviewer’s Comment:**

Figure 7 does not show new information or present information in a way which demonstrates new conclusions and could be moved to the supplementary figures or removed.

Author Response: The authors respectfully disagree with the recommendation to remove Figure 7. Although Figure 6 also shows data related to clinical chemistries, this is grouped data, with error bars representing the SEM. This graph is meant to show changes over time. Figure 7 more specifically looks at differences related to the magnitude of shifts for clinical chemistry measurements and shows individual data in relation to group means and SEMs. This type of analysis allows more meaningful conclusions to be drawn about actual differences between groups for the parameters assessed in relation to the magnitude of the response.

7. **Reviewer’s Comment**:

Discussion: Robust pharmacokinetic parameters were presented supporting chosen dosages. As the authors note, oral absorption of a drug may be altered during critical illness and/or by direct effect of the virus on tissue of the GI tract and/or organs involved in metabolism and excretion of the drug. The constraints of the ABSL-4 requirement for working with EBOV-infected animals make the decision to perform the pK studies in uninfected animals a sensible one. However, as the drug level measurement protocol was validated with approved inactivation steps for EBOV, if any stored samples from the challenge study are available which could be tested and compared with the predicted drug concentration at the time point sampled, this could provide insight into a possible mechanism of drug failure or viral breakthrough. As the authors discuss additional animal model testing (either higher dosing or IM/IP route), it would be reasonable to confirm the experimental plasma levels achieved if possible before progressing with additional animal testing.

Author Response: Thank you for this thoughtful recommendation. We agree that measuring plasma drug concentrations in EBOV-challenged animals could clarify whether altered exposure contributed to the observed lack of efficacy. We regret that insufficient stored sample volume from the current study prevents retrospective quantitation at relevant time points. To address this in future work, we will incorporate prospective PK sampling in infected animals and, if oral exposure appears unreliable during infection, prioritize alternative dosing strategies (e.g., higher doses or IM/IP routes).
